# Pre-treatment clinical features in central retinal vein occlusion that predict visual outcome following intravitreal ranibizumab

**DOI:** 10.1186/s12886-018-0701-x

**Published:** 2018-02-09

**Authors:** Kerr Brogan, Monica Precup, Amanda Rodger, David Young, David Francis Gilmour

**Affiliations:** 1 Glasgow Centre for Ophthalmic Research, Tennent Institute of Ophthalmology, Gartnavel General Hospital, Greater Glasgow and Clyde, Glasgow, UK; 2 Department of Ophthalmology, Gartnavel General Hospital, Greater Glasgow and Clyde, Glasgow, UK; 3 Department of Mathematics and Statistics, University of Strathclyde NHS, Greater Glasgow and Clyde, Glasgow, UK

**Keywords:** Retina, Central retinal vein occlusion, Anti-VEGF, Ranibuzimab

## Abstract

**Background:**

Predicting how patients with central retinal vein occlusion (CRVO) will respond to intravitreal anti-VEGF is challenging. The purpose of this study was to identify pre-treatment clinical features in CRVO that predict visual acuity (VA) following intravitreal ranibizumab.

**Methods:**

Medical records, fundus images and optical coherence tomography (OCT) scans of treatment naïve patients with CRVO receiving PRN intravitreal ranibizumab were retrospectively reviewed. Early Treatment Diabetic Retinopathy Study (ETDRS) VA and central retinal thickness (CRT) were recorded at baseline, 3 and 12 months after starting therapy. Regression analysis was used to determine independent predictors of VA at 3 and 12 months follow-up. Possible predictors included baseline VA, age, presence of cotton wool spots (CWS), haemorrhages (few scattered or multiple deep), foveal detachment, CRT, time from presentation to treatment, number of injections given, presence of RAPD, and cause of CRVO.

**Results:**

Data from 52 eyes of 50 patients receiving intravitreal ranibizumab treatment for CRVO were analyzed. The mean pre-treatment VA was 43.3 (SD 22.5) letters, which improved to 52.0 (SD 24.3) letters at 3 months, then dropped to 42.0 (SD 30.26) at 12 months. Baseline CRT reduced from 616.7 μm (SD 272.4) to 346.0 μm (SD 205.2) at 3 months and 304.0 μm (SD 168.3) at 12 months. The following features were predictive of poorer VA after starting intravitreal ranibizumab: Poorer pretreatment VA (3-months, *P* = 0.010; 12-months, *P* = 0.006), increasing age (3-months, *P* = < 0.001; 12-months, *P* = 0.006), and presence of CWS (3-months, *P* < 0.001; 12-months, *P* = 0.045).

**Conclusion:**

Pre-treatment VA, older age, and presence of CWS are easily identifiable clinical features in the hospital setting which help predict visual outcome in patients with CRVO receiving intravitreal ranibizumab.

## Background

Central retinal vein occlusion (CRVO) is the second most common cause of visual impairment due to retinal vascular disease in the UK after diabetic retinopathy [1], with prevalence ranging from 0.1 to 0.5% [2, 3]. It occurs when there is thrombosis of the central retinal vein as it passes through the lamina cribosa of the optic nerve [2]. Optic disc oedema, increased dilatation and tortuosity of all retinal veins, widespread deep and superficial haemorrhages, cotton wool spots (CWS), and retinal oedema are classic clinical features of this condition. Visual deterioration in CRVO is due to a combination of macular oedema and macular ischaemia. The former results from ischaemic retina producing abnormally high levels of intra-ocular vascular endothelial growth factor (VEGF) leading to vascular leakage. Anti-VEGF treatments, including ranibizumab and aflibercept, have been shown to be safe and effective in large randomized controlled trials (RCTs) and have been approved by the regulatory authorities across the globe [3–6]. In more severe CRVO, however, there is greater retinal ischaemia and predicting clinical outcomes from intravitreal anti-VEGF therapy in these patients is challenging. The Royal College of Ophthalmologists (RCO) retinal vein occlusion guidelines describe a number of clinical features that can be used to assess the level of retinal ischaemia in CRVO. These include: poor visual acuity; presence of a relative afferent pupillary defect (RAPD); multiple dark deep intra-retinal haemorrhages; multiple CWS; retinal vein dilatation and tortuosity; fluorescein angiography (FFA) showing greater than 10 disc areas of retinal capillary non-perfusion on 7-field FFA; and electroretinogram (ERG) showing reduced b wave amplitude, reduced b:a ratio, and prolonged b-wave implicit time [1]. In the hospital service setting, however, ERG is not performed as part of routine practice and FFA carried out at presentation is often unreliable in detecting capillary non-perfusion due to masking from retinal haemorrhages. Furthermore, The RCO guidelines concluded that there was no evidence as to which clinical feature or combination of features best defines ischaemic CRVO.

The paucity of evidence for the treatment of severe ischaemic CRVO, together with the difficulties in accurately measuring levels of retinal ischaemia, make it difficult for the clinician to decide whether to treat patients presenting with CRVO on the severe end of the spectrum. The aim of this study was to identify clinical features of CRVO, readily available in the eye clinic at first presentation, which can be used to predict outcome following intravitreal anti-VEGF therapy.

## Methods

Clinical records of treatment naive patients with CRVO receiving a PRN treatment regime of intravitreal ranibizumab therapy from 2009 to 2015 across three hospitals in Glasgow were retrospectively reviewed. The Optical Coherence Tomography (OCT) and fundal images that were analyzed were captured using the TopCon OCT-2000 (Topcon, Tokyo, Japan). This is a standard imaging system which captures spectral domain OCT images and a 30 degree posterior pole colour fundus image.

**Fig. 1:**
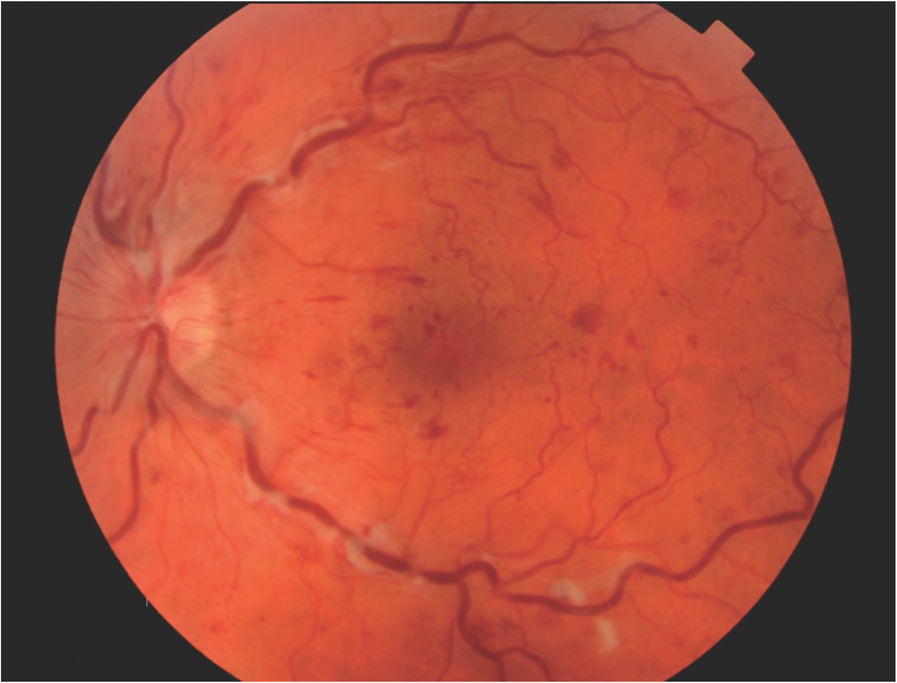
Fundus image from TopCon OCT-2000 showing cotton wool spots

**Fig. 2:**
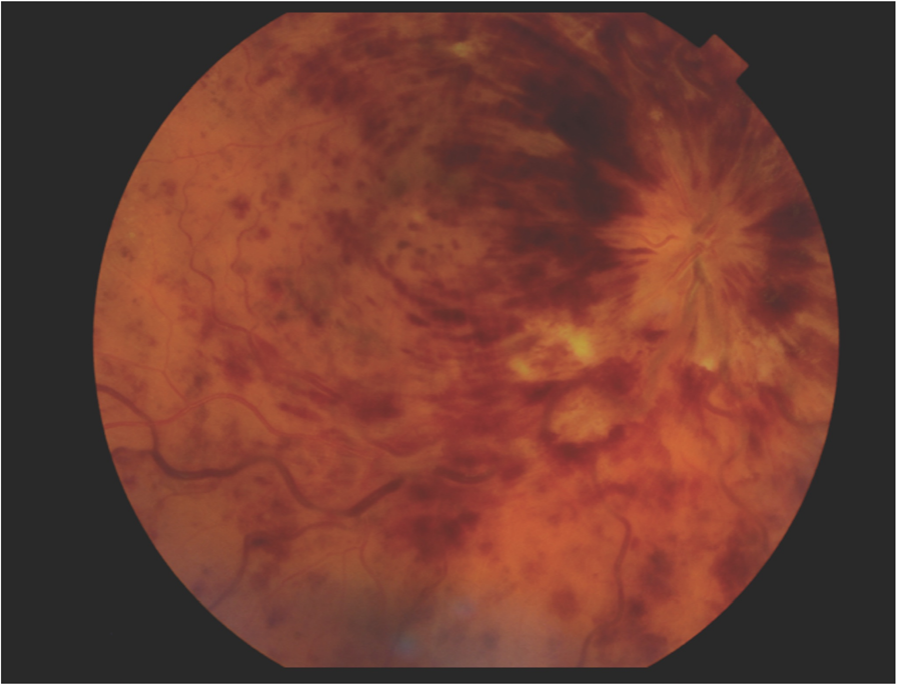
Fundus image from TopCon OCT-2000 showing multiple deep dark haemorrhages

**Fig. 3:**
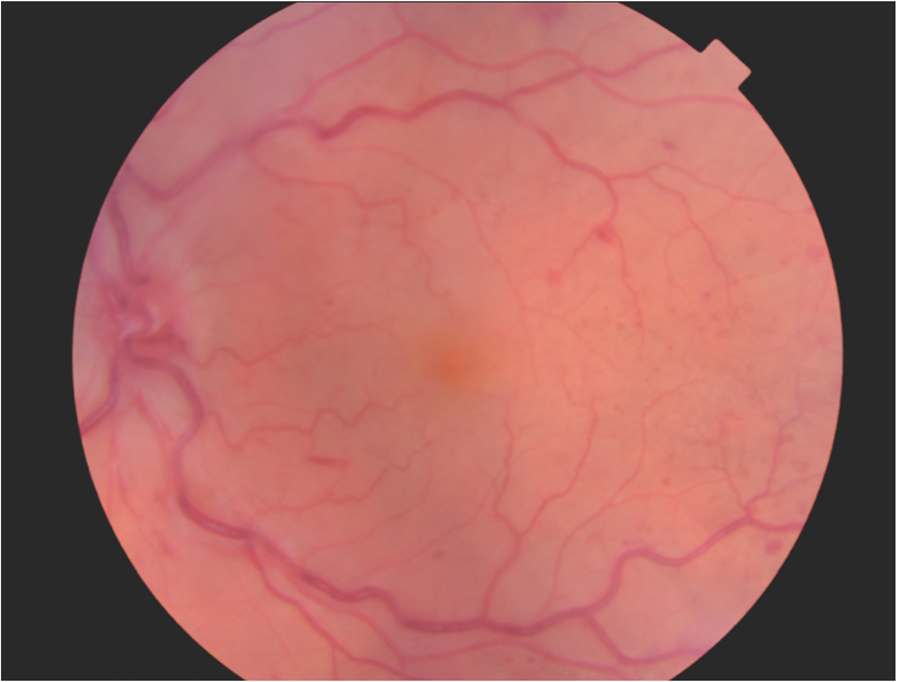
Fundus image from TopCon OCT-2000 showing few scattered haemorrhages

Regression analysis was used to determine independent predictors of ETDRS at 3 and 12 months after starting anti-VEGF therapy. Possible predictors were pre-treatment VA (ETDRS), age, presence of CWS (Fig. 1), presence of multiple deep retinal haemorrhages (Fig. 2) versus few scattered haemorrhages (Fig. 3), foveal detachment, central retinal thickness (CRT), length of time from presentation to first injection, number of injections, presence of RAPD and cause of CRVO. Variables which were significant in the final model at a 5% significance level were selected by backward regression analysis using Minitab (version 17). The majority of the VA measurements were recorded using Early Treatment Diabetic Retinopathy Study (ETDRS) letter score, however a small number were recorded in Snellen visual acuity. Snellen measurements were converted to ETDRS letter score using a recognized method [7].

Adhering to the West of Scotland Research Ethics Service review, ethics approval was not required for this study. This research has followed the Tenets of the Declaration of Helsinki.

## Results

Clinical data from 52 eyes of 50 patients who received intra-vitreal ranibizumab for CRVO were analyzed. Twenty three patients were female (46%) and 27 male (54%). The mean age at treatment was 72 years (SD 12.7). Forty-seven patients had a common vascular risk factor (hypertension, diabetes, hypercholesterolaemia or smoking). Of the remaining three patients without a recognized vascular risk, two had retinal vasculitis and one had co-existing ocular hypertension. Follow up was 100% at 3 months and 92% at 12 months (four patients were lost to follow up).

The mean pre-treatment VA was 43.3 (SD 22.5) ETDRS letters, which improved to 52.0 (SD 24.3) letters at 3 month, and dropped to 42.0 (SD 30.26) at 12 months after starting treatment. The mean number of intra-vitreal ranibuzimab injections was 2 (SD 1) at 3 months and 4 (SD 2) at 12 months. Nine cases (17.3%) had a pre-treatment VA of less than 20 letters (Snellen equivalent 3/60). The mean time from presentation to first injection was 29.2 days (SD 48.1, range 0–199 days). Eight eyes (15%) developed rubeosis over the course of treatment, three of which discontinued treatment at 3 months and seven by 12 months. CRT was available on OCT scan images in 42 of 52 (80.7%), 47 of 52 (90.4%), and 40 of 48 (83.3%) eyes at baseline, 3 and 12 months respectively. CRT reduced from 616.7 (SD 272.4) at baseline to 345.7 (SD 205.2) and 304.5 (SD 168.3) at 3 and 12 months respectively. Forty-five of 52 eyes (86.5%) had available OCT and clinical images of suitable quality for analysis of CWS, retinal haemorrhages and foveal detachment. Presence of RAPD was only available in clinical records of 8/52 cases. As the predominant cause of CRVO was common vascular risk factors (94.2%) this did not allow adequate comparison with other causes of CRVO in our data set. For these reasons presence of RAPD and cause of CRVO were not included in the regression analysis. Table 1 summarizes VA outcomes categorised by pretreatment clinical features.

**Table 1:** Mean VA at baseline, 3 and 12 months following ranibizumab in CRVO patients categorised by pre-treatment clinical features

Pre-treatment clinical features			Mean VA ± SD (ETDRS letters)		
		*N* (%)	Pre-Treatment	3 Months	12 Months
Base line VA (ETDRS letters)	> 59	14 (27%)	64.6 ± 6.0	64.1 ± 18.5	54.8 ± 26.3
	40–59	23 (44%)	49.8 ± 5.3	57.4 ± 17.0	45.2 ± 30.5
	20–39	6 (12%)	32.8 ± 6.6	36.3 ± 20.4	30.8 ± 18.5
	0–19	9 (17%)	0.6 ± 1.7	28.0 ± 31.4	21.2 ± 31.6
Age (years)	≥75	26 (50%)	40.3 ± 24.3	40.0 ± 24.5	24.7 ± 27.8
	< 75	26 (50%)	46.3 ± 20.5	63.3 ± 18.1	59.3 ± 21.8
CWS	Yes	26 (58%)	38.0 ± 25.5	40.8 ± 26.1	31.3 ± 30.4
	No	19 (42%)	48.2 ± 20.2	61.5 ± 17.1	49.6 ± 24.9
Haemorrhages	Multiple Deep Dark	29 (58%)	37.9 ± 25.7	45.8 ± 26.6	36.8 ± 30.7
	Few Scattered	19 (38%)	47.6 ± 19.4	53.0 ± 21.0	42.8 ± 28.1
	None	2 (4%)	60.0 ± 21.2	72.5 ± 17.7	27.5 ± 38.9
Foveal Detachment	Yes	28 (62%)	46.0 ± 22.9	48.4 ± 24.9	40.6 ± 28.5
	No	17 (38%)	38.2 ± 22.8	53.8 ± 21.7	37.4 ± 30.3

*VA* Visual acuity, *SD* Standard deviation, *ETDRS* Early Treatment Diabetic Retinopathy Study, *N* number of eyes, *CWS* cotton wool spots

Regression analysis showed that poorer baseline VA (*p* = 0.010), older age (*p* < 0.001) and presence of CWS (*p* < 0.001) predicted poorer VA at 3 months after starting intravitreal ranibizumab. Increasing age (*p* = 0.006) and poorer VA at 3 months (*p* < 0.001) predicted poorer VA at 12 months follow up. Univariate analysis showed presence of CWS to also be associated with poorer VA at 12 months (*P* = 0.045), however, this was less predictive than pre-treatment VA and age. Deep dark haemorrhages, foveal detachment, CRT, length of time from presentation to treatment, and number of ranibizumab injections received were not predictive of VA at 3 or 12 months follow up (Table 2).

**Table 2:** Univariate analysis of predictors of poorer VA at 3 and 12 months after starting intravitreal ranibizumab in patients with CRVO

	3 months (*P* value)	12 months (*P* value)
Poorer baseline VA	< 0.001	0.007
Increasing age	< 0.001	< 0.001
Presence of CWS	0.004	0.045
Deep dark haemorrhages	0.362	0.535
Foveal detachment	0.463	0.728
Increasing CRT	0.143	0.056
Time from presentation to IVT	0.199	0.529
Number of injections	0.859	0.949

*VA* visual acuity, *CRVO* central retinal vein occlusion, *CWS* cotton wool spots, *CRT* central retinal thickness, *IVT* intravitreal therapy

## Discussion

Intravitreal anti-VEGF therapy was first included in treatment regimes for macular oedema secondary to CRVO around 10 years ago [8]. Bevacizumab showed promising results correlating reduction in macular oedema with improved visual acuity [8, 9]. In 2007, ranibizumab was licenced by the European Medicines Agency (EMA) for use in RVO patients following the pivotal BRAVO and CRUISE clinical trials [3, 4]. Five years later aflibercept was licensed for CRVO following the COPERNICUS and GALILEO clinical trials [5, 6]. The benefits from treating non-ischaemic CRVO have been well documented in these studies, however, visual outcome in ischaemic CRVO is much harder to predict.

Accurately quantifying retinal ischaemia in CRVO is critical in the assessment of the disease. The methods of measuring ischaemia, however, are varied and not universally agreed. Hayreh et al. identified high-risk characteristics in CRVO patients who developed neovascularisation (NV) [10]. These criteria included best corrected visual acuity (BCVA) ≤ 6/60, loss of the 1-2e isopter on Goldmann visual field, RAPD ≥0.9 log units determined by neutral density filters, and electroretinogram reduction to ≤60% of corresponding A-wave. The Central Retinal Vein Occlusion Study (CVOS) group conducted the first major clinical trials to measure retinal ischaemia [11, 12]. Patients were deemed to be ischaemic if they had ≥10 disc areas of non-perfusion on FFA. These criteria were subsequently used in CRUISE, GALILEO and COPERNICUS [4–6] studies. Using 7-field fundus photography for FFA, however, only captures one third of the retinal surface. With the advent of wide field FFA, attempts have now been made to measure global retinal ischaemia in CRVO patients. Tsui et al. created the ischaemic index method, whereby areas of non-perfusion on wide field FFA were marked and expressed as a percentage of total visible retina [13]. Similarly, Nicholson et al. used concentric rings to measure topographic areas of non-perfusion on wide field FFA and correlated areas of non-perfusion with NV development [14]. Despite these advances, identifying ischaemic CRVO with FFA in the acute presentation of CRVO still has its challenges. Up to one third of patient’s FFAs are inadequately assessed due to masking from retinal haemorrhages or media opacities [10]. Isolated macular ischaemia may also be missed because assessment of the perifoveal vasculature is not possible in 11% of cases [15]. Additionally, retinal capillary closure can progress up to 3 months from onset of CRVO, therefore early FFA may underestimate the extent of retinal ischaemia at presentation [10, 15, 16]. ERG is an alternative method for measuring global ischaemia in CRVO [17]. Reduced b wave amplitude, reduced b:a ratio and prolonged b-wave implicit time are common findings that indicate retinal ischemia [18–21]. Although helpful in a research setting, ERG is not routinely carried out in all hospital settings. Visual fields (VF), on the other hand, are more readily available in the ophthalmology clinic, but not routinely used in assessment of CRVO patients. Hayreh et al. emphasized the importance of measuring peripheral retinal function with Goldman VF instead of focusing on VA alone [22]. VA, although being poor in global ischaemia, can also be reduced in isolated macular ischaemia. Trying to distinguish isolated macular ischaemia from global ischaemia in CRVO with the above investigations is important as the former carries a better prognosis [23]. Evidently, findings on FFA, ERG, and VF help identify retinal ischaemia, however, they are not always reliable and have to be analyzed in conjunction with clinical features. In recently published guidelines, the RCO concluded that there was no evidence as to which clinical feature or combination of features best defines ischaemic CRVO [1].

In this study we aimed to move away from the traditional classification of CRVO into ischaemic and non-ischaemic, and move towards identifying factors that can help predict the clinical outcome following anti-VEGF treatment. Longer duration of CRVO, poorer baseline VA, and older age have all been shown to predict poorer visual outcome in patients with CRVO undergoing anti-VEGF treatment [24–31]. Our study corroborates with the latter two features. To complement this literature, our study also found presence of CWS to be associated with poorer vision in CRVO patients undergoing anti-VEGF treatment. CWS are accumulation of cytoid bodies secondary to disruption to retinal axoplasmic flow, and tend to occur at the border of ischaemic retina [32]. If there are CWS at the posterior pole then there is likely to be a degree of adjacent retinal/macular ischaemia. CWS, along with baseline VA and patient age, are easily identifiable clinical features in CRVO which predict poorer response to treatment. Baseline and follow-up OCT images, however, also have a role in assessing visual prognosis. Increasing macular thickness and predominantly intra-retinal fluid (instead of sub-retinal fluid) are associated with poorer vision following anti-VEGF. Despite successful treatment of macular oedema, if there is persistent disruption of external limiting membrane and ellipsoid zone, visual gains are significantly poorer [26]. On the other hand, if there is preserved foveal depression on OCT then visual outcome is more promising [33]. In our study we looked at foveal detachment as a potential prognostic factor in CRVO, however, we found no significant correlation with VA after treatment. Another important feature when assessing OCT scans of CRVO patients is choroid thickness; increased choroidal thickness predicts better functional response from anti-VEGF [29]. Clearly there is a collection of prognostic clinical and OCT features of CRVO emerging in the literature. In clinical practice, these should be actively looked for when considering anti-VEGF treatment.

When treating CRVO with intravitreal ranibuzimab, the CRUISE study suggests that we can expect almost 50% of patients to gain 15 ETDRS letters or greater at 6 months follow up [4]. So why are our results not to living up this standard? Our study included CRVO patients with a full range of disease severity, including patients who later developed neovascularisation. Our broader phenotypic range of patients, although having poorer visual gains, may be more representative of real world data. Wai et al. reported similar short term results in their real world study with an average letter gain of 9.6 letters at 6 months compared to 8.7 letters at 3 months in our cohort [31]. At 12 months, however, our average VA drops to 42 ETDRS letters, which is 1.3 letters below average baseline VA. The eight patients (15% of our cohort) included in our analysis who became rubeotic at 12 months may account for the drop in average VA. Another contributing factor is that we may have been under-treating our patients. Our average number of ranibuzimab injections at 12 months was 4 (SD 2). This is slightly lower than six injections reported by Wai et al. [31]. In the CRUISE study, however, patients were consistently receiving monthly injections [4]. Our patients were treated on a PRN basis, and in our earlier use of anti-VEGF some patients did not receive 3 monthly injections as a loading dose. This, in conjunction with patients not adhering to strict monthly follow up in the NHS setting may explain the lower injection rate and inefficiency of ranibuzimab. On the other hand, number of injections in our data analysis did not significantly influence VA in our cohort.

If a patient with CRVO is thought to have significant retinal ischaemia, should we still be offering anti-VEGF treatment? The RCO guidelines state that macular oedema in eyes with ischaemic CRVO should be treated in the same way as those with non-ischaemic CRVO [1]. With little supporting published evidence in this area, treatment of ischaemic CRVO patients remains controversial. The RAVE study set out to assess whether ranibizumab could alter the natural history of CRVO in high risk eyes with three out of four Hayreh criteria [34]. Twenty patients received monthly ranibizumab for 9 months followed by 3 months of observation then monthly PRN injections for 24 months. Nine patients completed the full 36 months of treatment. The mean ETDRS letters gained was 21.4. As the patient numbers were small in this study and there was a large range of visual change following treatment (− 23 to + 74 letters), the secondary outcome finding should be viewed with caution. With regards to the primary outcome measure, this study demonstrated that NV formation was only delayed and not prevented [34]. The question on whether to treat patients with global retinal ischaemia has also been addressed in two recent reviews. Ford et al. concluded that the treatment benefits of intravitreal anti-VEGF therapy are only likely to apply to patients with non-ischaemic CRVO and that there are no proven treatments available for established ischaemic CRVO [35]. This was further corroborated by Gerding et al. who stated that ranibizumab is generally not recommended for patients presenting with irreversible ischaemic visual loss because of the limited availability of phase 3 trial data in this patient population [36]. This review did, however, recommend considering ranibuzimab when macular oedema is present in perfused retinal areas adjacent to macular ischaemic retinal zones, because some patients may experience a benefit. Based on our study and recently published data, younger patients are more likely to gain vision following anti-VEGF in ischaemic CRVO, and should therefore be offered treatment more readily [27].

Patients with CRVO are a heterogenous group and clinical outcomes, particularly in patients with more severe CRVO, are extremely difficult to predict. Instead of classifying patients into ischaemic and non-ischaemic groups, our study focuses on identifying clinical features in CRVO that predict poorer visual outcome from intravitreal anti-VEGF therapy. Our results complement the current knowledge base in this field and will help guide patients and doctors when deciding on whether to commence treatment. We hope other researchers will use our methodology for future studies to uncover other clinical features that may be important.

## Conclusion

Poorer pre-treatment VA, older age, and presence of CWS on standard 30 degree fundus photography predict poorer visual outcomes in patients with macular oedema secondary to CRVO receiving intravitreal ranibizumab. This information can be used during the consultation to help council patients on the likelihood of achieving good visual recovery if they opt for treatment.

## Abbreviations

BCVA: Best corrected visual acuity
CRT: Central retinal thickness
CRVO: Central retinal vein occlusion
CWS: Cotton wool spots
ERG: Electroretinogram
ETDRS: Early Treatment Diabetic Retinopathy Study
FFA: Fundus fluorescein angiography
NV: Neovascularisation
OCT: Optical Coherence Tomography
RAPD: Relative afferent pupillary defect
VA: Visual acuity
VEGF: Vaso-endothelial growth factor
VF: Visual fields
